# Type 2 diabetes-induced neuronal pathology in the piriform cortex of the rat is reversed by the GLP-1 receptor agonist exendin-4

**DOI:** 10.18632/oncotarget.6823

**Published:** 2016-01-05

**Authors:** Grazyna Lietzau, Thomas Nyström, Claes-Göran Östenson, Vladimer Darsalia, Cesare Patrone

**Affiliations:** ^1^ Karolinska Institutet, Department of Clinical Science and Education, Södersjukhuset, Internal Medicine, Stockholm, Sweden; ^2^ Karolinska Institutet, Department of Molecular Medicine and Surgery, Stockholm, Sweden; ^3^ Medical University of Gdansk, Department of Anatomy and Neurobiology, Gdansk, Poland

**Keywords:** diabetes, GLP-1R, Goto-Kakizaki rats, olfaction, piriform cortex

## Abstract

Type 2 diabetes (T2D) patients often present olfactory dysfunction. However, the histopathological basis behind this has not been previously shown. Since the piriform cortex plays a crucial role in olfaction, we hypothesize that pathological changes in this brain area can occur in T2D patients along aging. Thus, we determined potential neuropathology in the piriform cortex of T2D rats, along aging. Furthermore, we determined the potential therapeutic role of the glucagon-like peptide-1 receptor (GLP1-R) agonist exendin-4 to counteract the identified T2D-induced neuropathology.

Young-adult and middle-aged T2D Goto-Kakizaki rats were compared to age-matched Wistars. Additional Goto-Kakizaki rats were treated for six weeks with exendin-4/vehicle before sacrifice. Potential T2D-induced neuropathology was assessed by quantifying NeuN-positive neurons and Calbindin-D28k-positive interneurons by immunohistochemistry and stereology methods. We also quantitatively measured Calbindin-D28k neuronal morphology and JNK phosphorylation-mediated cellular stress. PI3K/AKT signalling was assessed by immunohistochemistry, and potential apoptosis by TUNEL.

We show T2D-induced neuronal pathology in the piriform cortex along aging, characterized by atypical nuclear NeuN staining and increased JNK phosphorylation, without apoptosis. We also demonstrate the specific vulnerability of Calbindin-D28k interneurons. Finally, chronic treatment with exendin-4 substantially reversed the identified neuronal pathology in correlation with decreased JNK and increased AKT phosphorylation.

Our results reveal the histopathological basis to explain T2D olfactory dysfunction. We also show that the identified T2D-neuropathology can be counteracted by GLP-1R activation supporting recent research promoting the use of GLP-1R agonists against brain diseases. Whether the identified neuropathology could represent an early hallmark of cognitive decline in T2D remains to be determined.

## INTRODUCTION

Type 2 diabetes (T2D) is one of the most prevalent chronic diseases in the modern world and its incidence is expected to rise substantially in the upcoming years [1]. Accordingly to the American Diabetic Association (http://www.diabetes.org/in-my-community/awareness-programs/older-adults/), approximately 25% of Americans over the age of 60 years have diabetes, and aging of the U.S. population is widely acknowledged as one of the drivers of the diabetes epidemic.

Previous studies showed that T2D patients have increased odor detection threshold [2], decreased odor-identification ability [3, 4], and increased risk of anosmia [5]. Recent studies confirmed a relationship between T2D and olfactory dysfunction as well as the correlation between lower olfactory scores and the presence of diabetic complications [6, 7]. Moreover, a recent pre-clinical work has demonstrated loss of olfactory sensory neurons with accompanying reduced olfactory discrimination in mice after exposure to a high-fat diet [8]. In addition, in the same model of T2D as used in our study (Goto-Kakizaki rats), a significantly increased glucose utilization in the olfactory bulb has been shown to result in increased taste aversion [9], in which olfactory cues play a determinant role [10].

Insulin resistance and insulin deficiency, which are two hallmarks of T2D at different stages, could be important contributing factors in the development of T2D-induced olfactory dysfunction and growing evidence for the role of insulin in the modulation of olfaction has been recently reported [11]. Obese, insulin-resistant rats have a decreased level of tyrosine-phosphorylated proteins in the olfactory bulb and piriform cortex [12]. Furthermore, insulin binding in the olfactory bulb of these rats is decreased [13].

Despite the fact that these results clearly show olfactory dysfunction in T2D, a specific neuropathology responsible for this has not been previously reported.

The piriform cortex (PC) is the largest of the olfactory cortical areas that receives direct synaptic input from the mitral and tufted cells of the olfactory bulb *via* the lateral olfactory tract. This evolutionary old, three-laminar *paleocortex* is critical for perception of odors [14] since neurons located in this brain area play a crucial role in odor coding (anterior part of the PC encodes odor identity, whereas posterior – odor quality) [15]. There is also growing evidence for the involvement of interneurons in synaptic inhibition in the PC after olfactory stimulation and recent electrophysiological studies showed that interneurons in the PC tend to be broadly excited by a range of different odors [16, 17]. The coded signals in the PC are further transmitted to other CNS regions [14], since this brain area is synaptically connected with the endopiriform nucleus, the anterior olfactory nucleus, olfactory tubercle, and cortical amygdala [14].

We hypothesize that olfactory dysfunction in T2D has to be linked to a specific brain neuropathology on histological and quantitative level. Since the PC is a key area involved in the regulation of olfaction, the present work aimed to identify potential neuronal pathology in the PC of T2D rats along aging.

Glucagon-like peptide-1 (GLP-1) is an incretin hormone, which enhances glucose-dependent insulin secretion *via* a specific G-protein-coupled GLP-1 receptor (GLP-1R) [18]. However, GLP-1 has a very short half-life due to rapid degradation. Exendin-4 (Ex-4) is a stable synthetic form of GLP-1. For these properties, it has been developed in clinical use for the treatment of T2D [18, 19]. Besides its anti-diabetic properties, Ex-4 can cross the blood brain barrier [20] and preclinical work supports neuroprotective role of Ex-4 and other GLP-1R analogues in several neurological disorders (reviewed in [21-23]). Thus, another goal of this study was to determine the potential efficacy of Ex-4 against T2D-induced neuronal pathology in the PC.

## RESULTS

### Glycaemia and insulin deficiency are increased by aging in GK rats

GK and Wistar rats at 3 and 13 months of age have been monitored for fasted blood glucose and plasma insulin levels. 3-month-old GK rats showed slightly, but significantly higher fasting glycaemia as compared to Wistar rats (~9mM *versus* ~6mM), while 13-month-old GK rats showed very high levels of hyperglycaemia (~18mM). Plasma insulin levels were significantly lower in GK rats already at 3 months as compared to age-matched Wistar controls (~2μg/L *versus* ~4μg/L). At 13 months the insulin levels decreased even further in GK rats (less than 1μg/L). The glycaemic data of Study 1 are presented in our recent publication [24].

**Figure 1:**
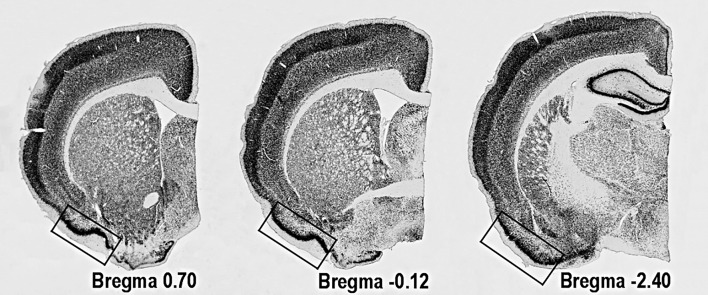
Location of PC on coronal sections of rat's brain The areas used for quantitative analyses are marked with the rectangle.

### T2D induces atypical NeuN nuclear staining in the piriform cortex

**Figure 2:**
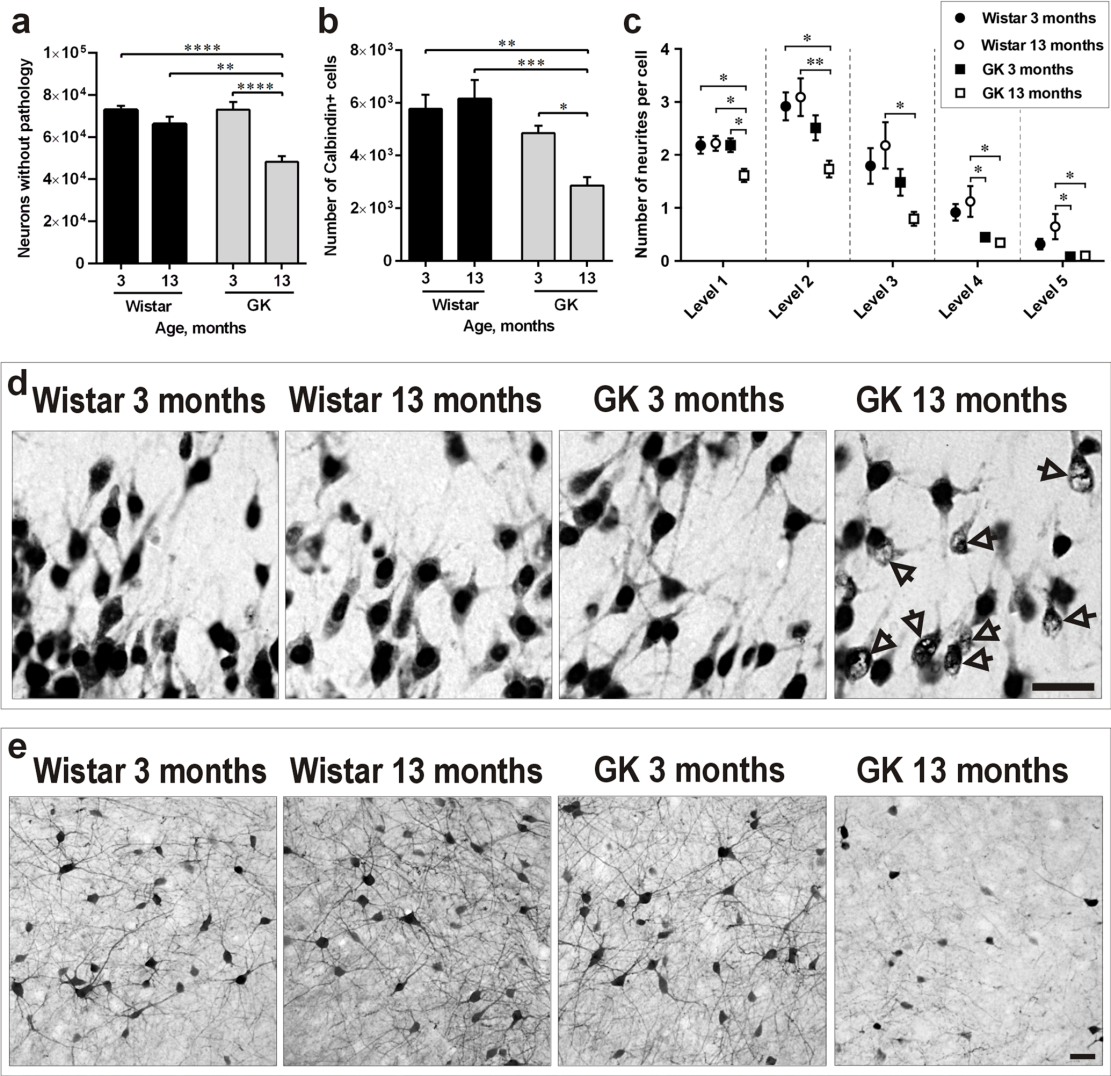
Diabetes induces neuronal pathology in the PC along aging **a**., **b**., **c**. Number of NeuN-positive neurons presenting normal morphology **a**., number of Calbindin-D28k-positive interneurons **b**. and of their neurites **c**. in the PC of 3- and 13-month-old Wistar and GK rats. **d**. Representative photomicrographs demonstrating the morphology of NeuN-positive neurons. Arrows indicate neurons with abnormal NeuN immunoreactivity. **e**. Representative photomicrographs demonstrating morphology of Calbindin-D28k-positive cells with visibly reduced neuronal branching and soma size in 13-month-old GK rats. Scale bars 50μm. Bars indicate means ± SEM. *P < 0.05, **P < 0.01, ***P < 0.001, ****P < 0.0001 (*n* = 6-7).

Sections from 3- and 13-month-old GK and Wistar rats' brains were stained for the specific neuronal marker NeuN. Similarly to our previous work in the isocortex [24], morphological observations identified a significant proportion of NeuN-positive neurons with abnormal NeuN distribution and large portions of the nucleus being negative for NeuN staining in the PC of 13-month-old GK rats (Figure 2d). This effect was not observed in the other three experimental groups. In order to quantify this T2D-induced effect, we counted only NeuN-positive neurons presenting normal morphology in the four experimental groups. The PC of 13-month-old GK rats contained approximately 30% less normal-looking NeuN-positive neurons than the PC of 3-month-old GK (*P*<0.0001) and 3- or 13-month-old Wistar rats (*P* <0.0001 and *P*<0.01, respectively; Figure 2a).

### γ-aminobutyric acid (GABA) neurons expressing Calbindin-D28k are negatively impacted by T2D

Presence of GABAergic interneurons expressing different calcium binding proteins has been previously shown in the PC, suggesting their important role in the plasticity of this region [25]. Since interneurons positive for Calbindin-D28k are crucial in the pathogenesis of T2D-related central nervous system complications such as dementia and Alzheimer's disease (AD) [26, 27], we determined whether this neuronal subpopulation in the PC was negatively impacted by T2D. The results depicted in Figure 2b revealed a not statistically significant trend towards the reduction of Calbindin-D28k-positive neurons already in 3-month-old GK rats *versus* 3- or 13-month-old Wistars. However, the PC of 13-month-old GK rats contained a significantly lower number of Calbindin-D28k-positive neurons in comparison to 3-month-old GK rats (*P*<0.05) as well as to 3- and 13-month-old Wistars (*P*<0.01 and *P*<0.001, respectively). In addition, stereological quantification (Figure 2c) as well as visual observations (Figure 2e) identified a substantial proportion of Calbindin-D28k-positive neurons with a dramatic reduction in neuronal branching in 13-month-old GK rats in comparison to the other three experimental groups.

### T2D induces cellular stress, but not neuronal death in the piriform cortex

**Figure 3:**
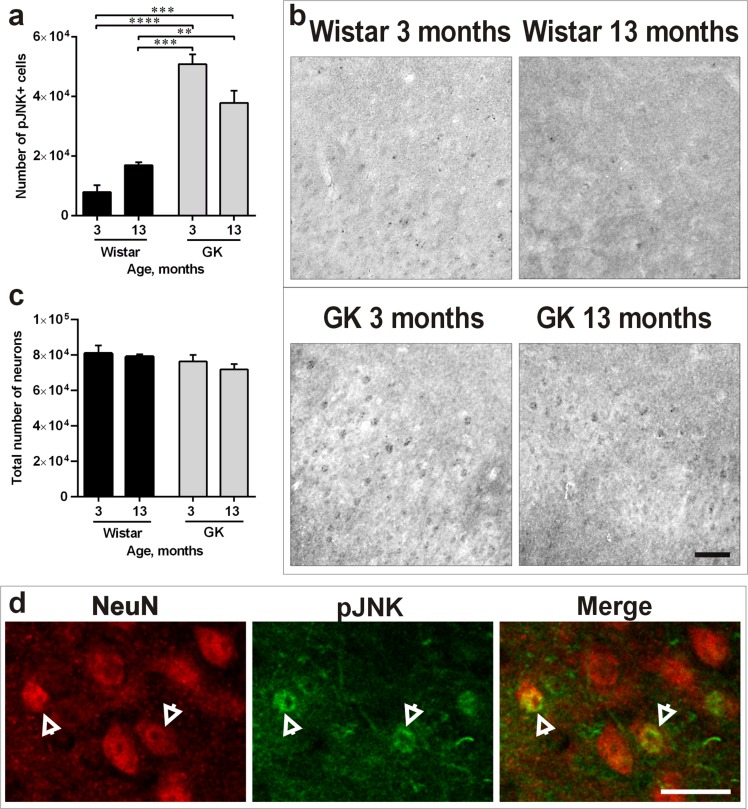
Diabetes induces cellular stress, but doesn't lead to neuronal death in the PC **a**., **b**. Number of pJNK-positive cells (a) and representative photomicrographs demonstrating increased JNK phosphorylation (b) in 3- and 13-months-old GK rats *versus* Wistars. **c**. Total number of neurons (presenting both normal and abnormal morphology) in the four experimental groups. **d**. representative photomicrographs of NeuN-positive neurons with increased expression of pJNK in the PC of 13-month-old GK rats. Arrows indicate double-stained cells. Scale bars 100μm. Bars indicate means ± SEM. **P < 0.01, ***P < 0.001, ****P < 0.0001 (*n* = 6-7).

We determined potential presence of apoptosis by TUNEL and the potential presence of cellular stress in the PC by quantifying the phosphorylated form of Jun-N-terminal kinase (JNK). TUNEL analysis showed no sign of apoptosis in the four animal groups (data not shown). However, JNK phosphorylation was enhanced in T2D rats *versus* Wistar controls in both 3-month-old (*P*<0.0001) and 13-month-old GK rats (*P*<0.01; Figure 3a). Representative photomicrographs presenting this effect are shown in Figure 3b. To rule out the possibility that the T2D effect on the PC led to neuronal death, quantifications of the total number of neurons (NeuN-positive neurons presenting both normal and abnormal morphology) were performed. The results showed no difference in the total number of neurons confirming neuronal pathology rather than neuronal death in 13-month-old T2D rats (Figure 3c). Double-staining of NeuN/pJNK confirmed the presence of neuronal stress in the PC (Figure 3d).

### Ex-4 reduces hyperglycaemia and increases insulin secretion in GK rats

**Figure 4:**
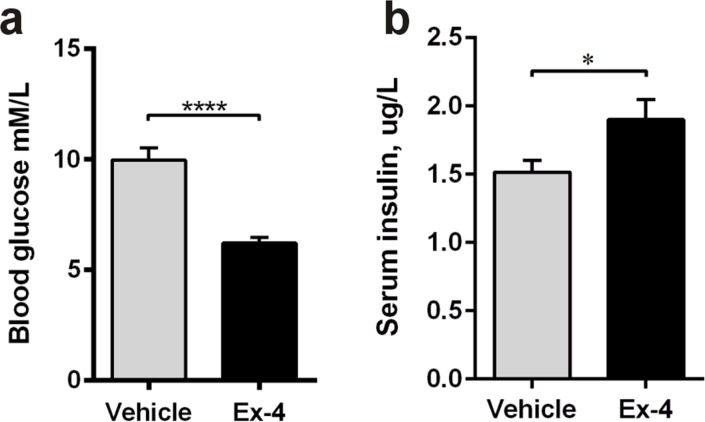
Ex-4 reduces hyperglycaemia and increases insulin secretion in GK rats Fasted blood glucose **a**. and fed serum insulin **b**. concentrations in 9-months-old vehicle- (*n* = 8) and Ex-4-treated (*n* = 10) GK rats. Bars indicate means ± SEM. *P < 0.05, ****P < 0.0001.

In Study 2, 9-month-old GK rats were treated with Ex-4 or vehicle for 6 weeks before sacrifice. Ex-4 significantly decreased blood glucose (p<0.0001; Figure 4a) and increased insulin secretion (*P*<0.05; Figure 4b).

### T2D-induced neuropathology in the piriform cortex is counteracted by chronic Ex-4 treatment

**Figure 5:**
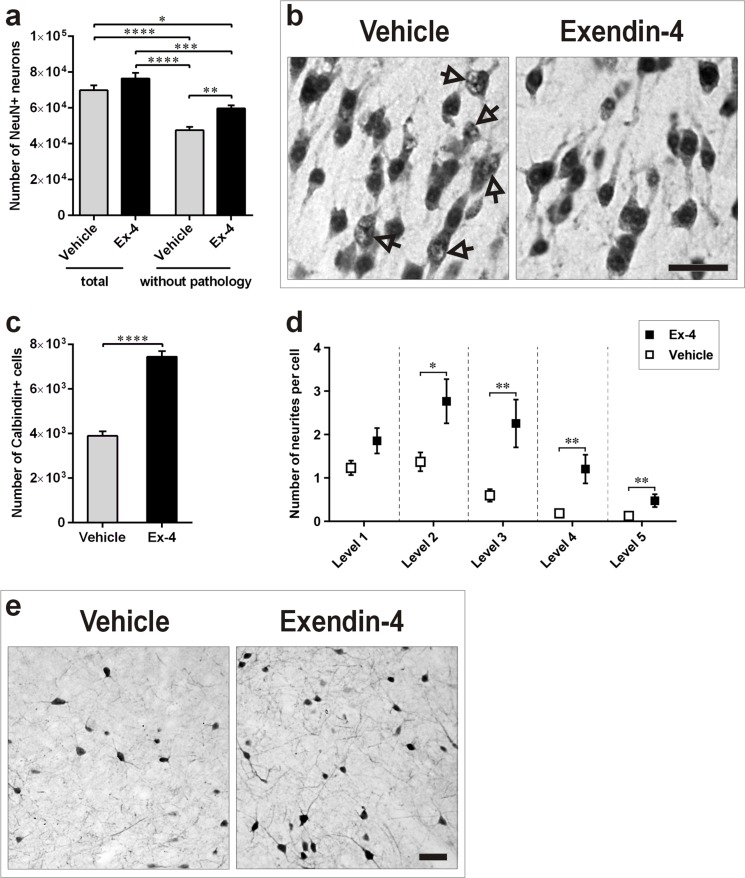
Ex-4 counteracts neuronal pathology in the PC of GK rats **a**., **b**. Number of total and normal/healthy NeuN-positive neurons (a) and representative photomicrographs demonstrating their morphology (b) in the PC of 9-months-old GK vehicle- (*n* = 8) and Ex-4-treated (*n* = 10) GK rats. Arrows indicate neurons with abnormal NeuN immunoreactivity. **c**., **d**., **e**. Number of Calbindin-D28k-positive interneurons (c) and number of their neurites (d) in control and Ex-4-treated GK rats, and representative photomicrographs showing the Ex-4 effect (e). Scale bars 50 μm. Bars indicate means ± SEM. *P < 0.05, ***P < 0.001, ****P < 0.0001.

A pilot study revealed that morphological features of the above described neuronal pathology in 13-months-old GK rats were already present at 9-months of age. Thus, to determine the potential role of Ex-4 in counteracting the identified neuronal pathology induced by T2D in the PC, quantification of NeuN-positive neurons without pathology was performed in 9-month-old GK rats treated for 6 weeks with Ex-4 or vehicle. Ex-4 significantly increased the number of normal/healthy neurons in comparison with vehicle-treated GK rats (*P*<0.01; Figure 5a). The Ex-4-mediated effect is also clearly reflected in the photomicrographs depicted in Figure 5b. We did not find changes in the total number of neurons (NeuN-positive neurons presenting both normal and abnormal morphology) in the PC of 9-months-old GK rats following 6-weeks of Ex-4 treatment (Figure 5a).

### Ex-4 exerts neurotrophic effects in Calbindin-D28k-positive neurons in the piriform cortex

To determine the potential role of Ex-4 to counteract the decreased number of Calbindin-D28k-positive neurons induced by T2D in the PC, quantification of Calbindin-D28k-positive neurons was performed in 9-month-old GK rats treated for 6 weeks with Ex-4 or vehicle. Ex-4 remarkably increased the number of Calbindin-D28k-positive neurons in comparison with vehicle-treated GK rats (Figure 5c; *P*<0.0001). The effect of Ex-4 seemed to be neurotrophic as visible by increased cell body and larger arborization of neurites (Figure 5e). Stereological counting of the number of neuronal neurites up to the fifth level confirmed our visual observations (Figure 5d).

### Ex-4 treatment decreases T2D-induced cellular stress in the piriform cortex

**Figure 6:**
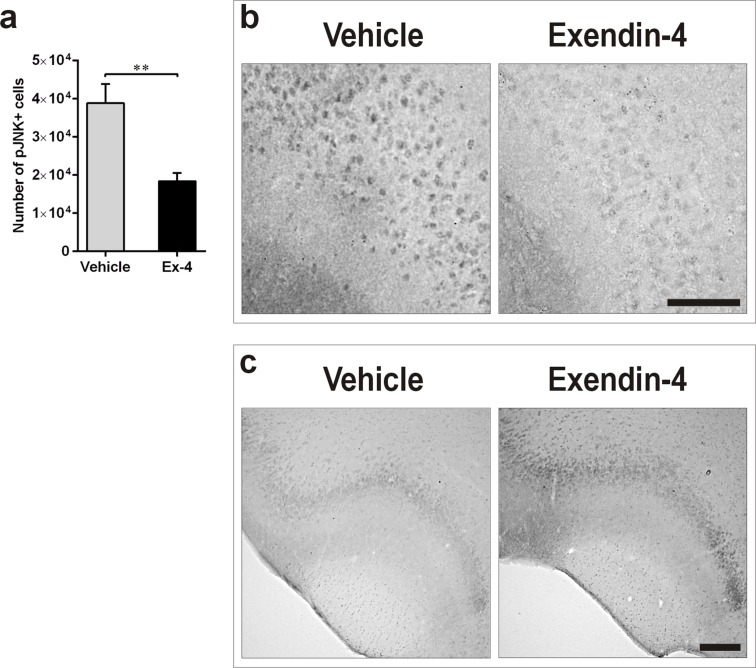
Ex-4 decreases T2D-induced cellular stress in the PC of GK rats **a**., **b**. Number of pJNK-positive cells in the PC of 9-months-old vehicle- (*n* = 8) and Ex-4-treated (*n* = 10) GK rats (a) and representative photomicrographs showing the effect (b). Scale bar 150 μm. **c**. Representative photomicrographs illustrating the increased phosphorylation of AKT in response to Ex-4 in the PC of GK rats. Scale bar 400μm. Bars indicate means ± SEM; **P < 0.01.

In order to understand whether the neurotrophic effect mediated by Ex-4 in the T2D PC correlated with decreased cellular stress, we also quantified the number of cells positive for the phosphorylated form of JNK by IHC. Ex-4 decreased JNK phosphorylation in 9-month-old GK rats in comparison with vehicle (Figure 6a, *P*<0.01). Representative photomicrographs demonstrating this effect are presented in Figure 6b.

### The neurotrophic effect of Ex-4 correlates with increased AKT phosphorylation

The phosphatidylinositol 3-kinase (PI3K)/AKT signalling pathway plays an important role in the regulation of neuronal function [28, 29]. To determine whether the Ex-4-mediated neurotrophic effect correlated with increased activation of this pathway, we quantified the phosphorylated form of AKT by IHC in the PC of vehicle- and Ex-4-treated GK rats. Six weeks of Ex-4 treatment moderately increased AKT phosphorylation in T2D rats (Figure 6c).

## DISCUSSION

Our results show T2D-induced neuropathology in the PC characterized by atypical nuclear morphology and increased cellular stress. We also demonstrate the specific vulnerability of Calbindin-D28k-positive interneurons to T2D. This effect was significantly counteracted by the treatment with the GLP-1R agonist Ex-4.

Several studies have reported a relationship between T2D and olfactory dysfunction (see Intro) leading to the hypothesis that this brain impairment has to be linked to a specific brain neuropathology on histological and quantitative level. The PC is the largest cortical region that receives direct synaptic input from the olfactory bulb, which in turn receives direct input from the olfactory epithelium, thus being a critical brain area for the perception of odors [14]. By showing neuropathology in the PC of the T2D brain, to our knowledge, this is the first report to provide a histopathological basis to explain olfactory dysfunction in T2D.

In this study, a morphological and quantitative neuronal analysis of healthy, pre-diabetic and overtly T2D rats of different ages identified atypical/abnormal nuclear appearance of neurons in the PC of overtly T2D rats, as assessed by NeuN staining. Similar findings have been recently shown in the isocortex of T2D GK rats [24]. NeuN is an intrinsic component of the neuronal nuclear matrix which is present in the majority of mature neurons in the brain [30]. The precise role of NeuN is not known, although it has been shown to be a regulator of neuronal-specific splicing [30]. Thus, although abnormal NeuN staining in the PC of GK rats cannot prove a specific and functionally relevant negative effect of T2D in this brain area, the results strongly point out to a potential unhealthy state of these neurons in GK rats.

To identify which neuronal population was mainly targeted by the T2D pathology, we focused our studies on the calcium-binding protein Calbindin-D28k positive GABAergic interneurons. Disrupted calcium homeostasis has been reported in the brains of AD patients [31] and recent works have shown the crucial role of Calbindin-D28k in the pathogenesis of AD [27] as well as its neuroprotective role against brain ischemia [32]. Furthermore, the vulnerability of other subtypes of interneurons involved in calcium homeostasis has been shown in the PC of AD patients [33]. Our results show a dramatic decrease (~ 50%) of Calbindin-D28k-positive neurons in the PC of the middle-aged T2D rats *versus* age-matched Wistar rats. However, this effect is likely to be the result of Calbindin-D28k down-regulation rather than neuronal loss. This conclusion is supported by three main evidences in the current study: 1) the count of NeuN-positive neurons presenting both normal and atypical morphology did not reveal any difference in total cell number among the four groups indicating absence of neuronal loss 2) we recorded no sign of apoptosis by TUNEL (data not shown), and 3) the middle-age T2D rats showed Calbindin-D28k-positive neurons with diminished neurite arborization and perikaryon size. Altogether these observations support a pathological state of Calbindin-D28k-positive neurons. This hypothesis is also supported by the results showing cellular stress by increased JNK phosphorylation in T2D rats already at 3 months of age (when hyperglycaemia starts rising [24]) suggesting that the T2D-iduced brain pathology begins at early stages although it does not lead to neuronal loss and apoptosis, at least until the rats are middle-aged (13-months-old). It remains to be determined whether prolonged cellular stress will lead to cell death at later stages of the diabetic pathology.

There are several obvious underlying reasons to explain the reported detrimental effect of T2D in the PC including hyperglycaemia, dyslipidemia, microvascular disease and dysfunctional insulin signalling. In addition, inflammation could play an important role causing the identified neuronal pathology [34]. Interestingly, our recent data in the isocortex of GK rats showed increased microglial activation [24].

In Study 2 we show that the identified T2D-induced neuronal pathology in the PC of 9-month old GK rats was remarkably counteracted by 6 weeks of treatment with the GLP-1R agonist Ex-4. Our pilot studies revealed that GK rats had developed the identified neuropathology already at 9 months. Therefore, in the second experiment the intervention with Ex-4 started at 9 months of age. We administered 0.1 μg/kg Ex-4 for 6 weeks to mimic a chronic treatment in T2D patients under a GLP-1R-mediated therapy. This dose of Ex-4 is given to T2D patients and it has been previously shown to be neuroprotective in several animal models [35]. The Ex-4 effect was likely neurotrophic since it led to a clear enhancement in neurite arborization and cellular body size of Calbindin-D28k-positive neurons without increasing the total NeuN neuronal count (sum of neurons both healthy and presenting pathological changes). Interestingly, regulatory effects of Ex-4 on the function of interneurons were recently reported by Korol S.V. et al. [36]. In addition, a trophic effect of Ex-4 on neuronal cells has also been previously shown *in vitro* by Perry et al. [37]. The data could be clinically relevant since Ex-4 is used for the treatment of T2D [38, 39] and it presents minimal side effects in humans. In addition, it can cross the blood-brain-barrier [20]. Finally, several studies have shown neurogenic and neuroprotective actions of Ex-4 and other GLP-1R agonists in rodent models of PD and AD [21, 22, 40].

To date, GLP-1R expression has not been reported in the rat PC although a thorough expression analysis of this brain area is lacking [41] and published results about the effectiveness of commercially available antibodies have been recently questioned [42]. However, preproglucagon positive neurons have been localized in this area [43] indicating potential GLP-1 expression. Thus, whether peripherally administered Ex-4 (as well as GLP-1 originating from preproglucagon positive neurons) activates GLP-1R in the PC, or if instead these peptides act *via* a GLP-1R-independent mechanism [44] remains to be determined.

It also remains to be determined whether the effect of Ex-4 is “directly” neurotrophic in the PC or if it is instead indirectly mediated by increased insulin and/or decreased glycaemia. In correlation with the neurotrophic effect of Ex-4 in the PC, we observed a moderate increase of AKT phosphorylation. AKT is a serine/threonine protein kinase, which is involved in many neuronal functions [28, 29]. Insulin is one of the main identified factors activating AKT phosphorylation. However, recent data has also shown that GLP-1R activation can directly trigger AKT phosphorylation, in correlation with neuroprotective effects [28]. Moreover, Xu W. et al. [29] reported a marked increase in phosphorylation of AKT in the brain of T2D rats after long-term Ex-4 treatment, in correlation with decreased AD pathology. Indeed glycaemia-independent neuroprotective effects caused by Ex-4 have been previously reported [45].

Patients with T2D are more prone to develop cognitive impairment, dementia and neurodegenerative diseases than healthy individuals [46-49], with consequent high medical and social costs, in addition to individual suffering. To reduce the onset or progression of T2D-induced neurological complications, it is therefore fundamental to identify early hallmarks of brain dysfunction in T2D. Interestingly, in addition to be related to T2D, olfactory dysfunction has also been shown to occur as an early manifestation (not always diagnosed on time) of neurodegenerative diseases such as PD and AD ([50, 51] and review: [52]). Furthermore, a recent clinical study has shown an association between olfactory dysfunction and decreased cognition in elderly T2D patients [53], leading to the hypothesis that olfactory dysfunction in T2D could represent an early marker for future cognitive impairment. Thus, although speculative, our data suggests that the identified neuronal pathology in the PC of the T2D brain could also represent an early hallmark of how T2D initiates the process of cognitive dysfunction and neurodegeneration.

In conclusion, we have identified a peculiar neuronal pathology in the PC of middle-aged T2D rats, which negatively impacts interneurons. We believe to have identified one important factor at the basis of olfactory dysfunction in T2D. We also showed that the identified neuropathology in the PC could be substantially counteracted by GLP-1R activation. Whether reversing the PC-related neuronal pathology in the T2D brain could represent a potential pharmacological target to treat olfactory dysfunction in diabetic patients or to even delay T2D-related brain complications remains to be determined.

## MATERIALS AND METHODS

### Animals and experimental groups

As an experimental model of T2D, we used male Goto-Kakizaki (GK) rats, which are Wistar-derived non-obese rats that spontaneously develop T2D [54]. GK rats are prone to peripheral neuropathy [55], behavioural impairments [56], loss of cerebral neurons along aging [24] and develop common T2D complications often observed in human patients [57].

### Normal and T2D rats were used in two studies

Study 1 (GK *versus* Wistar comparison). Four groups were analysed: 3-month-old GK (*n*=7), 13-month-old GK (*n*=6), 3-month-old Wistar (*n*=6), 13-month-old Wistar (*n*=6).

Study 2 (potential efficacy of Exendin-4 in GK rats). Two animal groups were analysed: vehicle-treated 9-month-old GK rats (*n*=8) *versus* Ex-4-treated 9-month-old GK rats (*n*=10). Ex-4 (0.1 μg/kg, *i.p.*) or PBS was given twice daily for 6 weeks before sacrifice.

Animals were housed on a 12 hours light/dark cycle with free access to food/water. Experiments were conducted in accordance with the Guidelines for Care and Use of Laboratory Animals published by U.S. National Institute of Health and approved by the local ethical committee.

### Glycemia and insulin measurements in Exendin-4 versus vehicle-treated GK rats

Animals were fasted for 6 hours and blood glucose was measured from tail-tip blood. The blood was also collected in fed state and serum insulin levels were determined by a rat insulin ELISA kit (kindly provided by Crystal Chem, Downers Grove, IL USA).

### Immunohistochemistry

Rats were deeply anesthetized and transcardially perfused with saline followed by 4% paraformaldehyde (PFA). The brains were extracted, cut in 40-μm-thick coronal sections and processed for immunohistochemistry as previously described [24]. The following primary antibodies were used: mouse anti-NeuN (1:300, Millipore, St. Charles, MO, USA), rabbit anti-Calbindin-D28k (1:1500, Abcam, Cambridge, UK), rabbit anti-phospho-SAPK/JNK (1:50, Cell Signaling Technology, Danvers, MA, USA), rabbit anti-phospho-AKT (1:25, Cell Signaling) and rabbit anti-GLP-1R (1:50, ab39072; Abcam). For chromogenic visualization, the ABC kit (Vector Laboratories, Burlingame, CA, USA) and 3,3′-diaminobenzidine (DAB, Sigma-Aldrich, St. Louis, MO, USA) were used. In fluorescent double-staining, AlexaFluor 594-conjugated secondary antibody (1:200; Life Technologies, Burlington, ON, Canada) and biotinylated secondary antibodies (1:200; Vector) followed by streptavidin-conjugated Alexa 488 (1:200; Life Technologies) were used. For apoptosis detection, DeadEnd Fluorometric TUNEL system (Promega, Madison, WI, USA) was used according to manufacturer's instructions.

### Quantitative analysis

Immunoreactive cells were counted using a computerized setup for stereology, equipped with NewCast software (Visiopharm, Hoersholm, Denmark), connected to Olympus BX51 epifluorescent/light microscope (Olympus, Tokyo, Japan). The number of NeuN-, Calbindin-D28k- and pJNK-positive cells in the PC was quantified on three evenly spaced sections (400μm) in each animal starting at 0.70mm from Bregma (Figure 1). Quantifications were performed using the optical fractionator method [24, 58]. Number of neurites in the Calbindin-D28k-positive cells was estimated in 50-60 randomly selected cells up to the fifth level of branching, e.g. the neurites protruding from cell body were marked as level 1 and every consecutive branching from the level 1 neurites - as level 2 and so on. The average number of neurites per cell was calculated.

### Statistical analysis

The data were analysed using GraphPad Prism 6 (CA, USA). The Student unpaired *t-test* or One-way ANOVA followed by the Turkey's *post-hoc* test were applied. Differences between the groups were considered statistically significant when *P*<0.05. Data are presented as means ± SEM.
